# Associations of Antibodies Against Several Infections With Alzheimer Disease Neuropathology: A Prospective Cohort Study Analysis

**DOI:** 10.1212/WNL.0000000000218584

**Published:** 2026-10-16

**Authors:** Caterina Felici, Rebecca E. Green, Charlotte Warren-Gash, Julia Butt, Tim Waterboer, Alun Hughes, Nishi Chaturvedi, Ashvini Keshavan, William Coath, Jonathan M. Schott, Marcus Richards, Dylan M. Williams

**Affiliations:** 1 Unit for Lifelong Health & Ageing, UCL, London, United Kingdom;; 2 Faculty of Epidemiology and Population Health, London School of Hygiene and Tropical Medicine, United Kingdom;; 3 Division of Infections and Cancer Epidemiology, German Cancer Research Center (DKFZ), Heidelberg, Germany;; 4 Dementia Research Centre, UCL, London, United Kingdom; and; 5 Division of Psychiatry, UCL, London, United Kingdom.

## Abstract

**Background and Objectives:**

Associations of common infections with Alzheimer disease (AD) risk have been reported. A hypothesized mechanism for these is cerebral β-amyloid (Aβ) aggregation as a defense to infection. We studied whether serologic measures of several infections in the UK population are associated with measures of AD neuropathology.

**Methods:**

This prospective association study used data from the 1946 British birth cohort. Circulating antibodies against 14 pathogens, measured at age 60–64 years, were modelled as pathogen serostatus (indicating lifetime exposure to an agent), pathogen burden indices (measuring cumulative exposure to 2+ pathogens), and seroreactivity tertiles (indicating recent immunologic activity against pathogens). Associations of these were tested with plasma phosphorylated tau at threonine 217 (p-tau217) (primary outcome) and Aβ status (secondary outcome), measured with PET scans, at ages 69–71 years. Modelling used multivariable quantile and logistic regression. Model 1 adjusted for sex and ages at serology and outcome assessment, and models 2 and 3 additionally adjusted for *APOE* ε4 carriage and education, respectively. We also tested for interactions in associations by *APOE* ε4 carriage, education, and sex, and for interactions between herpes simplex virus 1 exposure with cytomegalovirus (CMV) and varicella zoster virus (VZV) exposure.

**Results:**

A total of 1,356 and 424 individuals had complete data for p-tau217 and Aβ-PET analyses, respectively. Mean age at p-tau217 was 69.9 years (SD 0.7), and 51.3% of participants were female. Median p-tau217 was 0.30 pg/mL (interquartile range 0.21–0.44). 28.3% had Aβ-positive scans. No notable associations were observed for serology measures with either outcome in main models, except for an unexpected association of herpes simplex virus 2 seropositivity with lower p-tau217 at the 75th quantile (−0.07 pg/mL in seropositive individuals; 95% CI −0.10 to −0.03). Odds of Aβ-PET positivity may have been higher for individuals seropositive for Epstein-Barr virus (odds ratio [OR] 2.84; 95% CI 0.95–8.52) and *Toxoplasma gondii* (OR 1.69; 95% CI 0.94–3.05). There were also potential interactions in p-tau217 associations by *APOE* ε4 carriage (for *Helicobacter pylori* and CMV) and educational attainment (for *H pylori*).

**Discussion:**

These findings do not imply associations between exposures to many common infections and AD neuropathologies. The possibility of pathogen interactions with *APOE* ε4 carriage and education in relation to AD neuropathology warrants further study.

## Introduction

Besides established roles of some infectious agents in diseases of the CNS,^1^ there is increasing interest in whether infections affect dementia risk, particularly in relation to Alzheimer disease (AD).^2^ A major theory motivating research in this area is the “antimicrobial protection hypothesis,” proposing that specific pathogens in the CNS trigger aggregation of β-amyloid (Aβ) peptides as a defense mechanism.^3^ There might also be a greater role of overall infection burden rather than individual infectious agents in the development of AD.^4^ For instance, evidence suggests that varicella zoster virus (VZV) could be related to AD neuropathology by enhancing reactivation of herpes simplex virus 1 (HSV1).^5^ An interaction has also been observed between HSV1 and cytomegalovirus (CMV) infection in relation to AD risk.^6^ Since exposure to most herpesviruses and many other pathogens is common, yet only a subset of individuals develops clinical dementia, other factors might modify the relationship of pathogens with dementia. Some studies have reported that associations between common infections and cortical Aβ load, dementia, or cognition are more evident in individuals carrying *APOE* ε4 alleles^7-10^—the strongest genetic risk factor for late-onset AD—and in those with lower education.^11,12^ However, there is scant evidence available on the association of common infections—measured individually and in aggregate—with core subclinical AD neuropathology (Aβ and phosphorylated tau deposition), nor for potential modifiers of any such associations.^10,13-16^ To date, infections have been investigated in relation to the Aβ42/40 ratio in a small number of studies, and in relation to Aβ load measured by neuroimaging once, with results showing either no association or greater Aβ deposition.^10,13-16^

In this study, we examined prospective associations of evidence of exposure to 14 pathogens, individually and cumulatively, with measures of cerebral amyloidosis and tauopathy in a population-based birth cohort from the United Kingdom. We hypothesized that pathogen exposures would be associated with greater AD neuropathology and that associations would be more prominent in individuals carrying 1 or 2 *APOE* ε4 alleles and in individuals with lower education.

## Methods

### Study Population and Design

The Medical Research Council National Survey of Health and Development (NSHD; also known as the 1946 British birth cohort) is a population-based birth cohort that enrolled 5,362 individuals born across mainland Britain during 1 week in March 1946, using stratified sampling on social class.^17^ Participants have been followed continuously for 80 years to date. A subset of 502 NSHD participants was recruited into Insight 46, a neuroimaging substudy with initial assessments at ages 69–71 years to collect neuropsychological, neuroimaging, and blood biomarkers for dementia.^18^ This prospective association study principally uses data from (1) multiplex serology assays of plasma collected at ages 60–64 years in NSHD, (2) phosphorylated tau at threonine 217 (p-tau217) measures from plasma collected at home visits and clinical assessments at ages 69–71, and (3) Aβ-PET scans on individuals aged 69–71 years for the subset of participants in Insight 46 (Figure 1).

**Figure 1:**
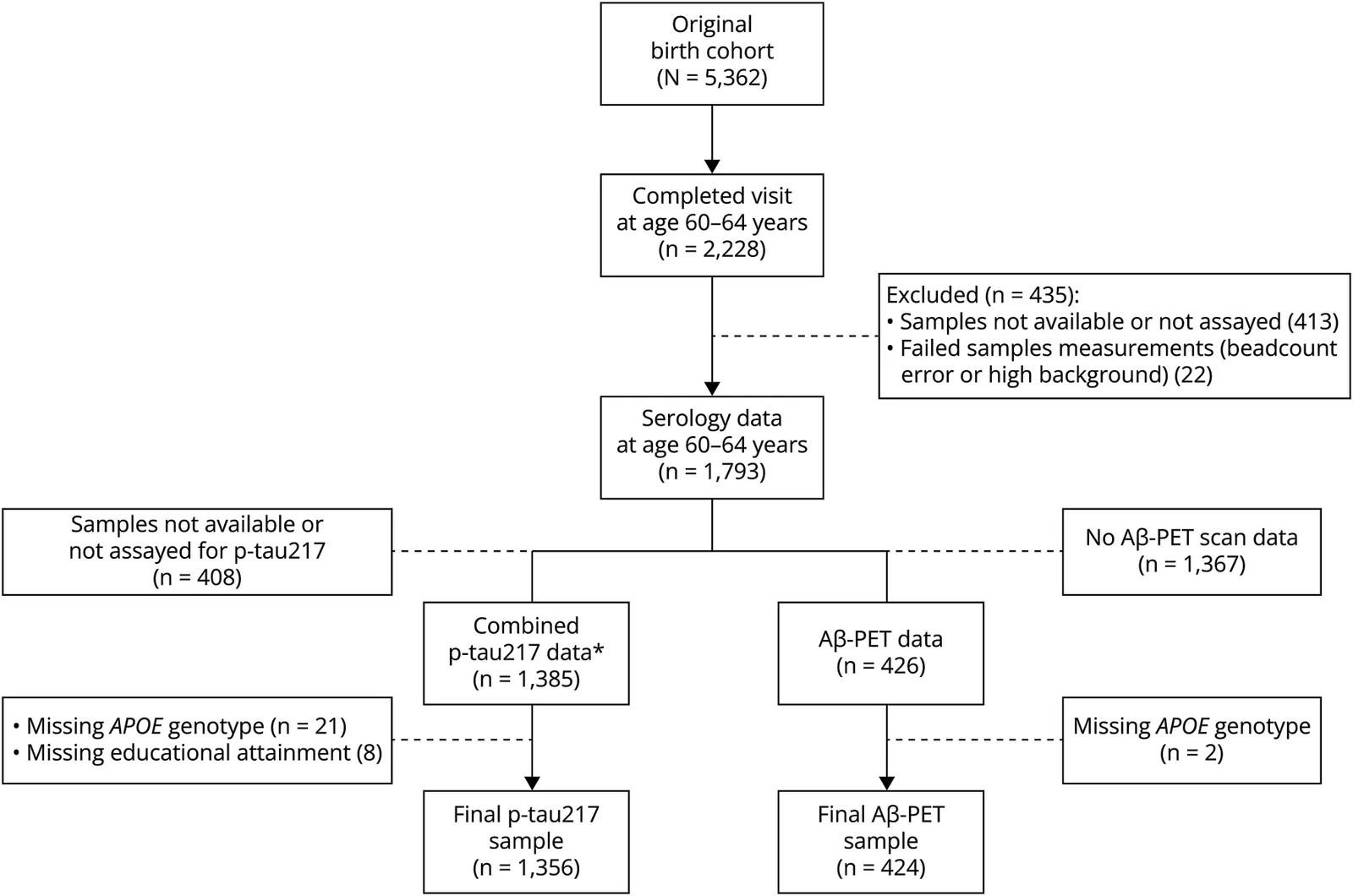
Study Flowchart Plasma p-tau217* from the NSHD home visits at age 69 (N = 952) were combined with p-tau217 data from Insight 46 participants (N = 484; ages 69–71)—see eMethods for more information. Aβ = β-amyloid; NSHD = National Survey of Health and Development; p-tau217 = phosphorylated tau at threonine 217.

### Assessment of Pathogen Exposures With Multiplex Serology

Exposure to pathogens was ascertained using a multiplex serology panel that quantifies circulating IgG antibody levels against antigens for different infectious agents, developed at the German Cancer Research Center in Heidelberg.^19,20^ We assayed 1,813 plasma samples collected between ages 60 and 64 years in 2006–2010^21^; 1,793 participants had valid serology data for 18 pathogens after quality control (Figure 1).

Serologic values were used to define (1) serostatus for individual pathogens, measured as a binary variable (seropositive/negative), indicating the presence or absence of detectable pathogen-specific antibodies and reflecting exposure at some point over the life course; (2) pathogen burden indices (PBIs) calculated as cumulative counts of serostatus variables; and (3) seroreactivity tertiles derived from the continuum of antibody concentrations, where higher concentrations are believed to reflect more recent infection or re-exposure, or more frequent reactivations of latent pathogens. Serostatus variables were derived using thresholds for a specific antigen or a combination of different antigens (eTable 1). Two PBIs were derived: one for the total number of infections and a neurotropic PBI restricted to a count of seropositive values for known neurotropic pathogens only (10 pathogens, denoted with an asterisk in eTable 1), which might be more relevant to AD pathogenesis.

For seroreactivity analyses, we included seropositive participants and only addressed antigens with at least 30 observations in each tertile to avoid very small sample sizes. This excluded all antigens for herpes simplex virus 2 (HSV2), *Toxoplasma gondii* (Tg), *Helicobacter pylori* (Hp), *Chlamydia trachomatis*, and some antigens for human herpesvirus 6A (HHV-6A) and 6B (HHV-6B) in analyses using Aβ-PET as an outcome, while 1 antigen for HHV-6A was excluded in analyses of p-tau217. We excluded *Human papillomaviruses* and Kaposi sarcoma–associated herpesvirus from all analyses because of low seropositivity (<5%). Risk of outcomes in higher vs lowest tertiles of antibody levels was compared in statistical models.

### P-Tau217 and Aβ-PET Measurements

Plasma p-tau217 was the primary outcome, given the large sample size available with these data and its high accuracy for detecting AD neuropathology measured by PET.^22^ This was assayed for home visit samples (N = 952) and for samples collected at clinic visits by Insight 46 participants (N = 484) using the ALZpath Simoa assay (Quanterix; absolute quantification yielding measurements in pg/mL).^22^ Assaying was undertaken at the UK DRI Biomarker Factory at UCL in separate batches for home visit and Insight 46 samples, with bridging between the batches. Further details are provided in eMethods.

Cerebral Aβ deposition, the secondary outcome, was measured by PET (Aβ-PET) using the 18F-Florbetapir PET ligand and quantified through the standardized uptake volume ratio (SUVR), with whole cerebellum used as a reference region following partial volume correction.^18^ SUVR values were converted to the Centiloid scale^23^ (0 Centiloid = mean SUVR in controls; 100 Centiloid = mean SUVR in AD). The value of 10 Centiloids was used as a threshold for amyloid positivity, considering a consensus view that cerebral amyloidosis can reliably be ruled out below 10 Centiloids.^24^ Aβ-PET reconstruction was performed using the pseudo-CT method.^18^ Pseudo-CT reconstructed values were imputed from the console-reconstructed values for 26 participants due to technical issues.

### Covariates

Covariates included age at serology, age at p-tau217 measurement, or age at neuroimaging; sex; *APOE* genotype; and highest educational attainment. *APOE* and educational attainment are both associated with AD risk and possibly with different susceptibility to infections.^25,26^ *APOE* genotypes were derived using variants rs7412 and rs429358 genotyped/imputed from genome-wide microarray data. A binary variable for *APOE* ε4 carriage was coded as ε4 carriers (ε4/ε4, ε3/ε4, ε2/ε4) and ε4 noncarriers (ε2/ε3, ε3/ε3 or ε2/ε2). A binary variable categorized educational attainment ordinary (O-level or equivalent or below) and advanced (A-Level or tertiary), based on self-report up to age 43 years.

### Statistical Analysis

Since this was an exploratory study, multiple testing correction was not performed. Complete-case analysis was performed because missing data (primarily for serology, p-tau, and amyloid values) were unlikely to be amenable to multiple imputation (and the extent of these missing data was limited; Figure 1). All statistical tests were 2-tailed at a significance level of 0.05. Analyses were performed in Stata 18 (StataCorp LLC, College Station, TX). Figures were generated in R, version 4.3.2.

#### Plasma P-Tau217 Modelling

Associations of serologic measures with p-tau217 were examined using quantile regression at the median and the 75th quantile. Quantile regression is robust to distributional assumptions and outliers. It also allows for testing of exposure-outcome associations at different parts of the outcome distribution, which may differ (for example, being more prominent at the tails)—especially for skewed outcomes, such as p-tau217.

We conducted 3 sets of models. Model 1 adjusted for sex, age at serology, and age at p-tau217 measurement. Model 2 adjusted for model 1 covariates plus *APOE* ε4 carriage. Model 3 adjusted for model 1 covariates plus education. All statistical models included sampling weights to adjust for the initial sampling strategy in the NSHD (individuals from manual social class families were underrepresented originally).^27^ For seroreactivity analyses, tertiles were modelled as ordinal variables; orthogonal polynomial contrasts were applied but did not support linear trends.

Two-way interaction terms were applied to model 1 to examine whether *APOE* ε4 carriage, education, or sex modifies associations of serologic measures with p-tau217. In addition, separate regression models incorporating interaction terms were used to test whether infection by VZV and CMV modifies the association of HSV1 serostatus with p-tau217, given previous suggestions of these interactions. Interactions were considered suggestive for *p* values less than 0.1. Interactions were tested only in median quantile regression. In analyses using seroreactivities tertiles as exposures, all interaction terms were tested simultaneously through a joint Wald test.

#### Aβ-PET Modelling

Secondary analyses for Aβ-PET followed the same methodology described above, with the following adaptations: (1) associations were examined through multivariable logistic regression; (2) model 1 was adjusted for sex, age at serology, and age at neuroimaging; and (3) interaction analyses were omitted because of reduced statistical power in the smaller sample. Linearity of continuous variable (ages at serology and neuroimaging) associations with log-odds of Aβ-PET positivity was assessed using fractional polynomials and quadratic terms, and indicated no requirements for nonlinear modelling.

### Sensitivity Analyses

In p-tau217 modelling, we conducted 3 sensitivity analyses. First, we repeated main analyses without applying sampling weights. Second, we tested the effects of further adjusting main analyses for body mass index (BMI) and estimated glomerular filtration rate (eGFR) to reduce noise in p-tau217 measurements because body size and kidney function may influence variability in p-tau217 concentration.^28,29^ Details of eGFR estimation are available in eMethods. Third, interaction terms were added to all models to assess whether relationships between serologic measures and p-tau217 differed by source of p-tau measurements (home visits or attendance at Insight 46).

For Aβ-PET modelling, we repeated main analyses: (1) without sampling weights, (2) excluding individuals with imputed data for Aβ-PET (N = 22), and (3) using a continuous measure of Centiloids as an outcome. Since Centiloids follow a gamma distribution, generalized linear models with a gamma log link were used. Centiloid values were adjusted to ensure positivity by adding the minimum value to all observations. Regression coefficients were multiplied by 100 to obtain relative percentage differences in the outcome per unit of exposure for ease of interpretation.^30^

To give context to association magnitudes from our modelling of the primary outcome, we also modelled associations of *APOE* ε4 carriage with p-tau217 with quantile regression at the median and 75th quantile, adjusting for age at p-tau217 measurement and sex.

### Standard Protocol Approvals, Registrations, and Patient Consents

Approvals for the relevant NSHD follow-ups were granted from the Central Manchester Research Ethics Committee, the Scotland A Research Ethics Committee, and the Queen Square Research Ethics Committee in London, United Kingdom (references 07/H1008/245, 08/MRE00/12, 14/SS/1009, 14/LO/1073, and 14/LO/1173). Participants gave informed, written consent for all follow-ups.

### Data Availability

NSHD data are available subject to approval by the cohort's Data Sharing Committee. For details, visit skylark.ucl.ac.uk/NSHD/

## Results

### Sample Characteristics

There were 1,356 participants with complete data for p-tau217 analyses and 424 for Aβ-PET analyses (Figure 1). Table 1 summarizes characteristics for both analytical samples. Compared with all individuals who attended the 60–64 year follow-up of the cohort, the p-tau217 and amyloid PET analytical samples had higher educational attainment but did not differ notably otherwise (eTables 2 and 3). Differences between the subsets of the primary analytical sample for plasma p-tau217 are displayed in eTable 4; participants with p-tau217 measured from home visits were slightly younger at sampling used for serology and p-tau217 measures and had a slightly higher proportion of women, a lower proportion of participants with higher educational attainment, and a higher median p-tau217.

**Table 1:** Characteristics of Each Analytical Sample

	P-tau217 sample (N = 1,356)	Aβ-PET sample^a^		
		Negative (N = 304, 71.7)	Positive (N = 120, 28.3)	Total (N = 424)
Age at serology, y, mean (SD)	63.2 (1.1)	63.4 (1.1)	63.3 (1.1)	63.4 (1.1)
Age at p-tau217, y, mean (SD)	69.9 (0.7)			
Age at neuroimaging, mean (SD)		70.7 (0.7)	70.6 (0.7)	70.6 (0.7)
Sex, n (%)				
Male	661 (48.7)	155 (51.0)	64 (53.3)	219 (51.7)
Female	695 (51.3)	149 (49.0)	56 (46.7)	205 (48.3)
*APOE* ε4 carriage, n (%)				
Noncarriers	945 (69.7)	246 (80.9)	57 (47.5)	303 (71.5)
Carriers	411 (30.3)	58 (19.1)	63 (52.5)	121 (28.5)
Highest educational attainment (up to age 43 y), n (%)				
Vocational or O-levels/equivalent or below	698 (51.5)	126 (41.4)	43 (35.8)	169 (39.9)
A-levels or higher	658 (48.5)	178 (58.6)	77 (64.2)	255 (60.1)
P-tau217, pg/mL, median (IQR)	0.30 (0.21–0.44)			
Centiloids, median (IQR)		−1.12 (−5.31 to 1.98)	33.64 (19.08–66.47)	1.00 (−3.50 to 14.76)

Abbreviations: Aβ = β-amyloid; IQR = interquartile range; p-tau217 = phosphorylated tau at threonine 217.

a Aβ status defined by Centiloid 10 cutoff.

Table 2 summarizes seroprevalences and PBIs for the analytical samples for p-tau217 and Aβ-PET analyses. Seroprevalences and PBIs for the larger sample of NSHD (and all of Insight 46) with serology data are available in eTable 5. There were no notable differences in serologic variables in the analytical samples vs all with serology data available. Seroprevalences for the 2 subsamples comprising the p-tau217 analytical sample separately are displayed in eTable 6. Serologic data were very similar between these subsets in all instances except Merkel Cell polyomavirus (MCV) serostatus (seropositivity was 8% higher in absolute terms in Insight 46).

**Table 2:** Seroprevalence in the Analytical Samples

	P-tau217 sample (N = 1,356)	Aβ-PET sample (N = 424)
Herpes simplex virus-1 seropositive, n (%)	907 (66.9)	274 (64.6)
Herpes simplex virus-2 seropositive, n (%)	103 (7.6)	26 (6.1)
Varicella zoster virus seropositive, n (%)	1,077 (79.4)	332 (78.3)
Epstein-Barr virus seropositive, n (%)	1,260 (92.9)	399 (94.1)
Cytomegalovirus seropositive, n (%)	725 (53.5)	223 (52.6)
Human herpesvirus-6a seropositive, n (%)	567 (41.8)	178 (42.0)
Human herpesvirus-6b seropositive, n (%)	730 (53.8)	218 (51.4)
Human herpesvirus-7 seropositive, n (%)	986 (72.7)	313 (73.8)
*Chlamydia trachomatis* seropositive, n (%)	238 (17.6)	59 (13.9)
*Toxoplasma gondii* seropositive, n (%)	334 (24.6)	102 (24.1)
*Helicobacter pylori* seropositive, n (%)	238 (17.6)	64 (15.1)
BK polyomavirus seropositive, n (%)	1,244 (91.7)	391 (92.2)
JC virus seropositive, n (%)	706 (52.1)	229 (54.0)
MC polyomavirus seropositive, n (%)	812 (59.9)	277 (65.3)
Human papillomavirus-16 seropositive, n (%)	40 (2.9)	12 (2.8)
Human papillomavirus-18 seropositive, n (%)	37 (2.7)	15 (3.5)
Total PBI, mean (SD)	7.3 (1.9)	7.3 (1.9)
Neurotropic PBI, mean (SD)	5.4 (1.7)	5.4 (1.7)

Abbreviations: Aβ = β-amyloid; PBI = pathogen burden index; p-tau217 = phosphorylated tau at threonine 217.

Kaposi sarcoma–associated virus was measured by the serologic assay but omitted from the table due to low cell counts for seropositive participants.

### Plasma P-Tau217

Results for the associations of pathogen serostatus and pathogen burden with p-tau217 are plotted in Figure 2 and presented numerically in eTable 7.

**Figure 2:**
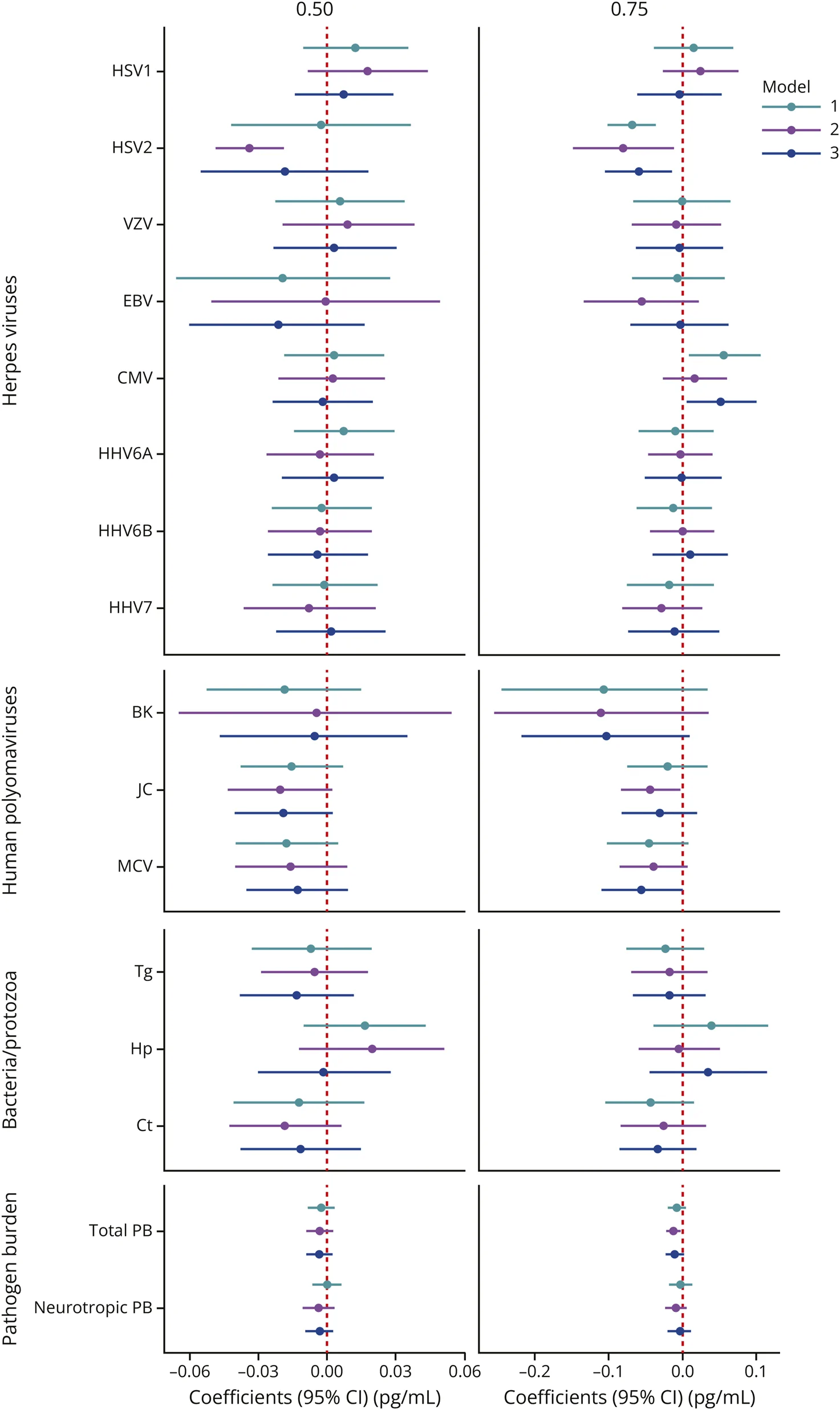
Associations of Pathogen Serostatus and Pathogen Burden With Plasma P-Tau217 Forest plots show weighted quantile regression results depicting differences (in mg/mL) in p-tau217 concentrations at the 50th (i.e., median) and 75th quantiles of the p-tau distribution per unit of exposure (left and right panels, respectively). Exposures were seropositive about seronegative for individual pathogens, or per 1 additional pathogen in the pathogen burden indices. Model 1 adjusted for sex, age at serology, and age at p-tau217 measurement. Model 2 adjusted for model 1 covariates plus *APOE* ε4 carriage. Model 3 adjusted for model 1 covariates plus education. BK = BK polyomavirus; CMV = cytomegalovirus; Ct = *Chlamydia trachomatis*; EBV = Epstein-Barr virus; HHV6A = human herpesvirus-6a; HHV6B = human herpesvirus-6b; HHV7 = human herpesvirus-7; Hp = *Helicobacter pylori*; HSV1 = herpes simplex virus-1; HSV2 = herpes simplex virus-2; JC = JC virus; MCV = Merkel cell polyomavirus; PB = pathogen burden; p-tau217 = phosphorylated tau at threonine 217; Tg = *Toxoplasma gondii*; VZV = varicella zoster virus.

For most pathogens, serostatus was not notably or consistently associated with higher concentrations of p-tau217 at either the median or 75th quantile of the p-tau distribution across model series. In most instances, p-tau differences by serostatus were modest, and CIs included the null. For a comparison of magnitudes, in this sample, *APOE* ε4 carriage was associated with 0.09 pg/mL higher p-tau217 (95% CI 0.05–0.12) at the median and 0.19 pg/mL higher p-tau217 (95% CI 0.12–0.26) at the 75th quantile (eTable 8). The largest positive point estimate for any pathogen serostatus at the median of the p-tau distribution was 0.02 pg/mL for Hp (model 1: 95% CI −0.01 to 0.04), and the highest at the 75th quantile was 0.06 pg/mL for CMV (model 1: 95% CI 0.01–0.11). Although this suggests p-tau217 concentration at the 75th quantile was higher in individuals seropositive for CMV, the magnitude was modest compared with *APOE* ε4's effect on p-tau217 concentration at the 75th quantile, and the association also attenuated when adjusted for *APOE* ε4 carriage. Seropositivity for HSV2 was associated with *lower* p-tau217 concentration at the 75th quantile across all models, while at the median in model 2 only (adjusted for *APOE* ε4 carriage). Results were close to the null for both overall and neurotropic PBIs.

Associations of seroreactivities with p-tau217 are presented in eFigures 1 and 2 and eTable 9. When examining associations with median p-tau217, lower p-tau217 concentration was observed in those with the highest tertile for antigen HHV6 IE1A (associations attenuated slightly after adjustment for ε4 carriage) and for the intermediate tertile of antigen HP 887/2 (vacuolating cytotoxin A) when adjusted for ε4 carriage and education, while higher p-tau217 concentration was observed for the intermediate tertile for antigen JC VP1. At the 75th quantile, the Epstein-Barr virus (EBV) Early Antigen-Diffuse (EAD) intermediate tertile was associated with lower p-tau217 concentration, while the HHV6 IE1B highest tertile was associated with higher p-tau217 concentration but attenuated when adjusted for ε4 carriage. There was indication of association of the Epstein-Barr Nuclear Antigen 1 (EBNA-1) peptide's highest tertile with lower p-tau217 concentration at 75th quantile, but this attenuated when adjusted for education.

### Interactions

We observed no interactions of HSV1 serostatus with either VZV or CMV serostatus in their associations with p-tau217 (eTable 10). There was suggestive evidence that associations of median p-tau217 with EBV, CMV, HHV6B, and Hp serostatus, and with some VZV, CMV, and Hp antigen seroreactivities, differed by *APOE* ε4 carriage (eTables 11 and 12). Suggestions for interactions by educational attainment were observed for Hp serostatus and some seroreactivity values for EBV, Hp, and MCV (eTables 11 and 12). No suggestive evidence for interactions by sex was observed in serostatus models, but there was some indication for seroreactivity values for Hp and MCV (eTables 11 and 12). There was no indication of notable interactions of either total or neurotropic PBIs with p-tau217 by *APOE*, education, or sex (eTable 11). Stratified results for *APOE* ε4 carriage are given in eTable 13. Median p-tau217 concentration was higher for individuals seropositive for CMV (by 0.08 pg/mL, 95% CI 0.01–0.14) and Hp (by 0.08 pg/mL, 95% CI 0.01–0.16) among *APOE* ε4 carriers only. Antigen CMV pp28 was associated with higher median p-tau217 concentration among *APOE* ε 4 carriers only. On the other hand, VZV antigen glycoprotein E / glycoprotein I (gE/gI) was associated with lower concentration of median p-tau217 at both tertiles in ε4 carriers only. Stratified results for education are presented in eTable 14. Hp serostatus was associated with opposing differences in median p-tau217 in those with lower education (0.04 pg/mL higher p-tau217; 95% CI 0.01–0.07) and higher education (−0.05 pg/mL p-tau217; 95% CI −0.09 to −0.01). Sex-stratified results for seroreactivity-p-tau217 associations for 2 Hp and MCV antigens are given in eTable 15.

### Aβ-PET

Associations of pathogen serostatus and pathogen burden with odds of a positive Aβ-PET scan are presented in eFigure 3 and eTable 16. There was a suggestion of higher odds of Aβ-PET positivity for individuals seropositive for EBV (model 1: odds ratio [OR] 2.84; 95% CI 0.95–8.52) and Tg (model 1: OR 1.69; 95% CI 0.94–3.05) with large point estimates, although considering the size of CIs, associations were estimated with low precision. To put association magnitudes in context, carriage of at least 1 ε4 allele associated with an OR of 5.16 for Aβ-PET positivity (95% CI 3.02–8.81) in this cohort.^31^ There was no evidence for associations of either PBIs or seroreactivity tertiles with Aβ-PET positivity (eFigures 3 and 4 and eTables 16 and 17).

### Sensitivity Analyses for Plasma P-Tau217

In analyses unweighted for the NSHD cohort's original sampling scheme, results resembled the main findings (eFigures 5–7). Analyses further adjusted for BMI and eGFR also produced similar results (eFigures 8–10).

When examining whether p-tau217 results differed by subsets of p-tau data (collected at home visits or Insight 46), there was evidence for interaction between source of p-tau data and HSV2 serostatus in relation to median p-tau217 in all 3 models and particularly when adjusted for *APOE* (*p* for interaction in model 2 <0.01; eTable 18). In stratified analyses, HSV2 serostatus showed evidence for association with lower median p-tau217 in home visit data only in model 2 (−0.06 pg/mL; 95% CI −0.08 to −0.04), with a point estimate in the opposite direction in Insight 46 data (0.03 pg/mL; 95% CI −0.01 to 0.08; eTable 19). There was a similar pattern of associations of Tg with p-tau217 by p-tau data collection source, albeit with less pronounced evidence for interaction (*p* = 0.10 in model 1; eTables 18 and 19). Several seroreactivity—p-tau217 associations also showed suggestive evidence of interaction by subsample (eTable 20), but not consistently across tertile results once stratified (eTable 21).

### Sensitivity Analyses for Aβ-PET

In unweighted analyses, most results resembled the main analyses except for EBV and Tg serostatus findings, which were both less precise and smaller in magnitude (model 1 OR for EBV 1.62 [95% CI 0.59–4.42]; model 1 OR for Tg 1.22 [95% CI 0.75–1.97]; model 2 and 3 results very similar for both). Results for pathogen serostatus, pathogen burden, and seroreactivities did not change notably when excluding participants with imputed Aβ-PET. There was evidence for higher levels of Aβ deposition among individuals seropositive for EBV when repeating the analyses using a continuous variable for Centiloids (model 1: 25% higher burden; 95% CI 0%–51%). Results for the associations of PBIs and seroreactivities with Aβ deposition also resembled the main analysis. Full data from these sensitivity analyses are available on request.

## Discussion

In this population-based cohort from the United Kingdom, we observed no marked evidence for associations of exposure to many common pathogens, measured by antibody levels, with AD neuropathology at ages 69–71 years. There were some suggestive findings when interactions by *APOE* ε4 carriage, educational attainment, and sex were considered—although these may have arisen by chance.

HSV1 has been investigated extensively in relation to AD, including for potential interactions with *APOE* ε4 carriage,^7,32,33^ and in 1 recent phase 2 trial of an antiherpetic medication administered to early AD cases who were seropositive for HSV1 or 2 (which yielded negative results).^34^ Our null findings are consistent with observations from other epidemiologic studies. One found associations of symptomatic herpesvirus infections with elevated plasma glial fibrillary acidic protein, a marker of astrogliosis, but not with plasma Aβ42/40 or neurofilament light chain.^14^ Other previous studies reported similarly null results for serologic measures of HSV and other common infections in relation to plasma Aβ.^15,16^ We previously found null or little evidence for associations of most pathogens under investigation here (including HSV1) with MRI-derived measures of dementia risk, including hippocampal volume.^21^ Another cohort study observed no associations of HSV, *Chlamydia pneumoniae*, Hp, and CMV serostatus with dementia incidence.^35^

There could be several reasons for null findings. First, the pathogens studied here may not be notable determinants of AD pathogenesis (at least as measured by the aggregation of AD's cardinal proteinopathies by age ∼70). Alternatively, defining infection exposure from IgG antibodies does not provide a measure of infection severity: it is possible that severe infections might influence AD pathogenesis more than milder ones. Another caveat is that infections could be involved in later stages of disease development after AD pathophysiology is established, rather than in preclinical phases.^36,37^ With AD neuropathology assessment at ∼70 years in this study, some participants with developing AD may not have been far along the disease trajectory. Last, exposure to several pathogens and interactions among them might be necessary for the development of neuropathology, rather than any individual infection. However, despite previously reported associations of PBIs with dementia risk and serum Aβ levels,^13^ we found no associations of PBIs or some key hypothesized pathogen interactions (HSV1 by VZV or CMV) with AD neuropathology measures here. We note that we would not have had sufficient statistical power to investigate the numerous possible 2-way (or higher order) interactions between other combinations of individual pathogens.

Among *APOE* ε4 carriers only, we observed higher median p-tau217 concentration in individuals seropositive for CMV and with higher antibody levels against CMV pp28 antigen, and in individuals seropositive for Hp. A greater risk of dementia in ε4 carriers infected by HSV1 and other pathogens (HIV, severe acute respiratory syndrome coronavirus 2) has been observed elsewhere.^25^ One study reported that ε4 carriers had higher levels of CMV IgG antibodies.^38^ There is conflicting evidence from studies that have examined CMV and Hp in relation to outcomes other than those in the present analysis (dementia, cognition, and neuroimaging), with evidence for and against interaction by ε4 carriage among these.^21,35,39^ Whether there might be a specific mechanism linking CMV or Hp to increased AD pathology in ε4 carriers alone, or whether these results might have arisen by chance, is unclear. Testing for these associations in comparable data elsewhere would help to clarify whether these are spurious.

Some of the findings—including lower p-tau217 among ε4 carriers with high antibodies against VZV antigen gE/gI —were unexpected, and existing research in relation to these is scant.^21,40^ Our observation of lower p-tau217 at the 75th quantile for individuals seropositive to HSV2 was also unanticipated, and the scant evidence related to this association to date would imply no AD risk difference or increased risk according to HSV2 exposure.^21,41^ Some antigen seroreactivities seemed to be variably and inconsistently associated with either higher or lower p-tau217 concentrations. We are not aware of prior evidence for sex interactions related to those we observed in p-tau217 models for Hp and MCV antigens (HP73 [UreA] and MCV VP1). Finally, there was a suggestion of positive associations between Hp serostatus and median p-tau217 concentrations for lower levels of education, while negative associations for higher levels of education (individuals with lower education seem to be, on average, more exposed to or susceptible to infections overall, and to Hp infection specifically).^11,42-44^ Each of these suggestive findings could be due to chance—particularly given the degree of multiple testing performed here—or from biases such as residual confounding.

In Aβ-PET analyses, our findings suggested the possibility of associations of EBV and Tg serostatus with Aβ deposition. Contrasting findings between serologic measures of infections with measures of amyloidosis and tauopathy have been observed elsewhere, where higher antibody levels for EBV EBNA-1, CMV, and HSV1 associated with lower Aβ42/40 (reflecting higher cortical amyloid) but to lower p-tau181 (reflecting lower amyloid-induced tauopathy).^45^ However, given the lack of these associations with p-tau217 (which is a strong predictor of Aβ status) in the larger sample with this outcome, along with associations of EBV and Tg with Aβ-PET closer to the null in sensitivity analyses where we omitted sampling weights, these findings may not be robust. Establishing whether these 2 pathogens associate with Aβ aggregation specifically beyond what is measured by plasma p-tau217 concentration would require further examination in other samples.

This study has several strengths. We measured exposure to many common infections through serology (i.e., not through self-reporting of clinically manifest infections) alongside large-scale data on accurate and specific biomarkers of AD pathology.^22^ Measurement of AD pathology with these biomarkers allows the study of AD neuropathology in a preclinical phase. The use of subclinical biomarkers as outcomes rather than a clinical endpoint for AD may reduce the influence of some biases, such as reverse causation and survival bias.

There are also several limitations. As an observational study, residual confounding can affect association estimates, although for this to explain our mostly null findings, we would need to have unaccounted factors strong enough to mask more prominent associations from being observed. NSHD participants with poorer health, worse socioeconomic circumstances, and lower childhood cognitive ability were underrepresented in the 69-year and Insight 46 assessments, producing the possibility of selection bias.^46,47^ If infections had increased study attrition due to heighted morbidity or mortality between exposure and outcome measurement, these exposures could also have affected selection. However, most of the infections being studied do not have common clinical manifestations among individuals aged 60–70 years, and we are unaware of data showing major increases in mortality risk for seropositive individuals in this age range.^48^ The lack of longitudinal serology and infection timing data limited temporal exposure characterization. Although we studied numerous infections which have been implicated in AD etiology previously, some could not be measured using the current multiplex serology platform, for example, *Chlamydia pneumoniae* and periodontal pathogens. Assaying of samples was in singlicate due to the scale of data generation, but duplicate measurements for internal control samples indicated low within-plate and cross-plate coefficients of variation. We did not have shingles vaccine data, but this vaccine was introduced in 2013 in the United Kingdom (after blood sampling used for these serology measures) and would not have influenced the VZV serology values. Moreover, NSHD participants became eligible for routine shingles vaccination at age 70 years in 2016—coinciding with the timeframe for p-tau217 and Aβ-PET measures. Hence, shingles vaccine uptake is unlikely to have influenced these outcomes either. Finally, since the NSHD comprises White British participants, generalizability to other populations may be limited.

Although these findings cast doubt on the roles of many common infections in the development of AD neuropathology, future research could help to establish whether more fine-grained characterization of infections (including timing, dose, and severity) or other infections not studied here is relevant to AD pathogenesis. Studying infections in relation to other biomarkers of neurodegenerative and neurovascular diseases including inflammatory responses could help to clarify potential effects of pathogens in other causes of dementia besides AD, and other AD-related biomarkers such as the tau microtubule-binding region containing residue 243 (MTBR-tau243) could be used to further examine the relevance of infections to later-stage AD pathogenesis.^49^ Finally, the generation of similar exposure data in large cohorts involving older participants with appreciable dementia incidence would help to test associations of pathogens individually and in combination with dementia risk directly. Other associations could be pertinent, considering evidence that suggests shingles vaccination (and potentially other vaccines) reduces dementia incidence,^50^ though not necessarily through an effect on AD pathogenesis specifically.

## Glossary

Aβ: β-amyloid
AD: Alzheimer disease
CMV: cytomegalovirus
EBV: Epstein-Barr virus
eGFR: estimated glomerular filtration rate
HHV-6A: human herpesvirus 6A
HHV-6B: human herpesvirus 6B
Hp: *Helicobacter pylori*
HSV1: herpes simplex virus 1
HSV2: herpes simplex virus 2
NSHD: National Survey of Health and Development
OR: odds ratio
PBI: pathogen burden index
p-tau217: phosphorylated tau at threonine 217
SUVR: standardized uptake volume ratio
Tg: *Toxoplasma gondii*
VZV: varicella zoster virus
